# Reply to Mahat, R.K.; Rathore, V. Comment on “Xiang et al. Association between the Triglyceride-Glucose Index and Vitamin D Status in Type 2 Diabetes Mellitus. *Nutrients* 2023, *15*, 639”

**DOI:** 10.3390/nu15184069

**Published:** 2023-09-20

**Authors:** Qunyan Xiang, Hui Xu, Junkun Zhan, Shuzhen Lu, Shuang Li, Yanjiao Wang, Yi Wang, Jieyu He, Yuqing Ni, Linsen Li, Yiyang Liu, Youshuo Liu

**Affiliations:** 1 Department of Geriatrics, The Second Xiangya Hospital of Central South University, Changsha 410011, China; bynsals55@csu.edu.cn (Q.X.); xuhui23@csu.edu.cn (H.X.); zhanjunkun@csu.edu.cn (J.Z.); ls330013@csu.edu.cn (S.L.); 503404@csu.edu.cn (Y.W.); 503503@csu.edu.cn (Y.W.); hejieyu@csu.edu.cn (J.H.); niyuqing@csu.edu.cn (Y.N.); 2204160117@csu.edu.cn (L.L.); liuyiyang@csu.edu.cn (Y.L.); 2 Institute of Aging and Age-Related Disease Research, Central South University, Changsha 410011, China; 3 Department of Nursing, Hunan Normal University School of Medicine, Changsha 410013, China; lushuzhen@hunnu.edu.cn

We are pleased to see that Mahat and Rathore [1] have commented on our paper studying the association between the triglyceride glucose index and vitamin D status in type 2 diabetes mellitus [2]. The authors have pointed out that the formula: triglyceride-glucose (TyG) index = Ln [fasting triglyceride (mg/dL) × fasting glucose (mg/dL)/2], was incorrect in our previous study [2]. They have also suggested using the following formula to calculate the TyG index values: Ln [fasting triglycerides (mg/dL) × fasting glucose (mg/dL)]/2. The reason for their concern was that the formula to calculate the TyG index was first proposed by Simental et al. in 2008 [3], which was consistent with that used in our study. However, Simental et al. clarified a mistake in the formula in 2020 and corrected it with another formula: Ln [fasting triglycerides (mg/dL) × fasting glucose (mg/dL)]/2 [4].

We read with great interest the article by Simental et al. [4]. However, the authors have not stated the reason for the correction of the formula. Further, we have searched a large number of literature, including some papers published in some influential journals, and found that most of them employed the following formula to calculate the TyG index: Ln [fasting triglyceride (mg/dL) × fasting glucose (mg/dL)/2] both before 2020 [5,6,7,8,9] and after 2020 [10,11,12,13,14,15,16]. However, some studies used the following formula to calculate the TyG index: Ln [fasting triglyceride (mg/dL) × fasting glucose (mg/dL)]/2 [17,18]. Currently, there is no consensus regarding which of them would be better to appropriately identify the presence of insulin resistance or vitamin D deficiency.

It made us feel confused about the use of the TyG index, and thus, we made a comparison of the TyG cutoff points between the two formulas using the same statistical settings. The results showed that TyG > 4.86 was determined as the optimal cutoff point for the identification of VDD when using the formula Ln [fasting triglycerides (mg/dL) × fasting glucose (mg/dL)]/2 to calculate the TyG index values, which was different from the cutoff point when using the formula Ln [fasting triglyceride (mg/dL) × fasting glucose (mg/dL)/2] to calculate the TyG index values. To our surprise, although the cutoff points were different, the sensitivity (75.0%) and specificity (41.9%) were the same regardless of which formula was used (Table 1).

To our knowledge, this study demonstrated for the first time that the two most commonly used formulas for calculating the TyG index have the same predictive value for the identification of vitamin D deficiency, although their cutoff points were different. The comparison of the TyG index using the two formulas in subsequent more large-scale studies may provide more evidence in choosing a more appropriate formula.

Thank you again for your interest in our article.

## Figures and Tables

**Table 1:** Comparison of the cutoff points of the TyG index calculated using different formulas for the identification of vitamin D deficiency.

	TyG 1	TyG 2
Cutoff points	9.03	4.86
Sensitivity	75.0%	75.0%
Specificity	41.9%	41.9%
AUC	0.647	0.647

The optimal cutoff points for the TyG index were determined using receiver operating characteristic (ROC) curve analysis. TyG 1 = Ln [fasting triglyceride (mg/dL) × fasting glucose (mg/dL)/2], TyG 2 = Ln [fasting triglyceride (mg/dL) × fasting glucose (mg/dL)]/2. AUC is the area under the curve.

## References

[1] Mahat R.K., Rathore V. (2023). Comment on Xiang et al. Association between the Triglyceride-Glucose Index and Vitamin D Status in Type 2 Diabetes Mellitus. *Nutrients* 2023, *15*, 639. Nutrients.

[2] Xiang Q., Xu H., Zhan J., Lu S., Li S., Wang Y., Wang Y., He J., Ni Y., Li L. (2023). Association between the Triglyceride-Glucose Index and Vitamin D Status in Type 2 Diabetes Mellitus. Nutrients.

[3] Simental-Mendia L.E., Rodriguez-Moran M., Guerrero-Romero F. (2008). The product of fasting glucose and triglycerides as surrogate for identifying insulin resistance in apparently healthy subjects. Metab. Syndr. Relat. Disord..

[4] Simental-Mendia L.E., Guerrero-Romero F. (2020). The correct formula for the triglycerides and glucose index. Eur. J. Pediatr..

[5] Park K., Ahn C.W., Lee S.B., Kang S., Nam J.S., Lee B.K., Kim J.H., Park J.S. (2019). Elevated TyG Index Predicts Progression of Coronary Artery Calcification. Diabetes Care.

[6] Luo E., Wang D., Yan G., Qiao Y., Liu B., Hou J., Tang C. (2019). High triglyceride-glucose index is associated with poor prognosis in patients with acute ST-elevation myocardial infarction after percutaneous coronary intervention. Cardiovasc. Diabetol..

[7] Su W.Y., Chen S.C., Huang Y.T., Huang J.C., Wu P.Y., Hsu W.H., Lee M.Y. (2019). Comparison of the Effects of Fasting Glucose, Hemoglobin A(1c), and Triglyceride-Glucose Index on Cardiovascular Events in Type 2 Diabetes Mellitus. Nutrients.

[8] Guerrero-Romero F., Simental-Mendia L.E., Gonzalez-Ortiz M., Martinez-Abundis E., Ramos-Zavala M.G., Hernandez-Gonzalez S.O., Jacques-Camarena O., Rodriguez-Moran M. (2010). The product of triglycerides and glucose, a simple measure of insulin sensitivity. Comparison with the euglycemic-hyperinsulinemic clamp. J. Clin. Endocrinol. Metab..

[9] Vizzari G., Sommariva M.C., Dei Cas M., Bertoli S., Vizzuso S., Radaelli G., Battezzati A., Paroni R., Verduci E. (2019). Circulating Salicylic Acid and Metabolic Profile after 1-Year Nutritional—Behavioral Intervention in Children with Obesity. Nutrients.

[10] Huang R., Lin Y., Ye X., Zhong X., Xie P., Li M., Zhuang X., Liao X. (2022). Triglyceride-glucose index in the development of heart failure and left ventricular dysfunction: Analysis of the ARIC study. Eur. J. Prev. Cardiol..

[11] Fritz J., Brozek W., Concin H., Nagel G., Kerschbaum J., Lhotta K., Ulmer H., Zitt E. (2021). The Triglyceride-Glucose Index and Obesity-Related Risk of End-Stage Kidney Disease in Austrian Adults. JAMA Netw. Open.

[12] Sajdeya O., Beran A., Mhanna M., Alharbi A., Burmeister C., Abuhelwa Z., Malhas S.E., Khader Y., Sayeh W., Assaly R. (2022). Triglyceride Glucose Index for the Prediction of Subclinical Atherosclerosis and Arterial Stiffness: A Meta-analysis of 37,780 Individuals. Curr. Probl. Cardiol..

[13] Huang R., Wang Z., Chen J., Bao X., Xu N., Guo S., Gu R., Wang W., Wei Z., Wang L. (2022). Prognostic value of triglyceride glucose (TyG) index in patients with acute decompensated heart failure. Cardiovasc. Diabetol..

[14] Han Y., Zhou Z., Zhang Y., Zhao G., Xu B. (2023). The Association of Surrogates of Insulin Resistance with Hyperuricemia among Middle-Aged and Older Individuals: A Population-Based Nationwide Cohort Study. Nutrients.

[15] Paublini H., Lopez Gonzalez A.A., Busquets-Cortes C., Tomas-Gil P., Riutord-Sbert P., Ramirez-Manent J.I. (2023). Relationship between Atherogenic Dyslipidaemia and Lipid Triad and Scales That Assess Insulin Resistance. Nutrients.

[16] Lee J.H., Kwon Y.J., Park K., Lee H.S., Park H.K., Han J.H., Ahn S.B. (2022). Metabolic Score for Insulin Resistance Is Inversely Related to Incident Advanced Liver Fibrosis in Patients with Non-Alcoholic Fatty Liver Disease. Nutrients.

[17] Simental-Mendia L.E., Ortega-Pacheco C.J., Garcia-Guerrero E., Sicsik-Aragon M.A., Guerrero-Romero F., Martinez-Aguilar G. (2021). The triglycerides and glucose index is strongly associated with hepatic steatosis in children with overweight or obesity. Eur. J. Pediatr..

[18] Simental-Mendia L.E., Sanchez-Garcia A., Guerrero-Romero F. (2023). Association of the triglycerides and glucose index and the homeostatic model assessment of insulin resistance with lipoprotein(a), apolipoprotein AI, and apolipoprotein B concentrations in children with normal-weight. Eur. J. Pediatr..

